# Design, Characterization, and First-In-Human Study of the Vascular Actions of a Novel Biased Apelin Receptor Agonist

**DOI:** 10.1161/HYPERTENSIONAHA.114.05099

**Published:** 2015-03-11

**Authors:** Aimee L. Brame, Janet J. Maguire, Peiran Yang, Alex Dyson, Rubben Torella, Joseph Cheriyan, Mervyn Singer, Robert C. Glen, Ian B. Wilkinson, Anthony P. Davenport

**Affiliations:** From the Clinical Pharmacology Unit, University of Cambridge, Centre for Clinical Investigation, Addenbrooke’s Hospital, Cambridge, UK (A.L.B., J.J.M., P.Y., J.C., I.B.W., A.P.D.); Bloomsbury Institute of Intensive Care Medicine, University College London, London, UK (A.D., M.S.); and Unilever Centre for Molecular Sciences Informatics, Department of Chemistry, University of. Cambridge, Cambridge, UK (R.T., R.C.G.).

**Keywords:** β-arrestin G-protein coupled receptors, pulmonary arterial hypertension, [Pyr^1^]apelin-13

## Abstract

[Pyr^1^]apelin-13 is an endogenous vasodilator and inotrope but is downregulated in pulmonary hypertension and heart failure, making the apelin receptor an attractive therapeutic target. Agonists acting at the same G-protein–coupled receptor can be engineered to stabilize different conformational states and function as biased ligands, selectively stimulating either G-protein or β-arrestin pathways. We used molecular dynamics simulations of apelin/receptor interactions to design cyclic analogues and identified MM07 as a biased agonist. In β-arrestin and internalization assays (G-protein–independent), MM07 was 2 orders of magnitude less potent than [Pyr^1^]apelin-13. In a G-protein–dependent saphenous vein contraction assay, both peptides had comparable potency (pD_2_:[Pyr^1^]apelin-13 9.93±0.24; MM07 9.54±0.42) and maximum responses with a resulting bias for MM07 of ≈350- to 1300-fold for the G-protein pathway. In rats, systemic infusions of MM07 (10-100nmol) caused a dose-dependent increase in cardiac output that was significantly greater than the response to [Pyr^1^]apelin-13. Similarly, in human volunteers, MM07 produced a significant dose-dependent increase in forearm blood flow with a maximum dilatation double that is seen with [Pyr^1^]apelin-13. Additionally, repeated doses of MM07 produced reproducible increases in forearm blood flow. These responses are consistent with a more efficacious action of the biased agonist. In human hand vein, both peptides reversed an established norepinephrine constrictor response and significantly increased venous flow. Our results suggest that MM07 acting as a biased agonist at the apelin receptor can preferentially stimulate the G-protein pathway, which could translate to improved efficacy in the clinic by selectively stimulating vasodilatation and inotropic actions but avoiding activating detrimental β-arrestin–dependent pathways.

## Abstract 2

Supplemental Digital Content is available in the text.

The apelins are a family of peptides^1^ that have an emerging role in the human cardiovascular system with the most abundant isoform being [Pyr^1^]apelin-13. In healthy humans, the major action of infusion of apelin in the forearm was an increase in blood flow caused by the release of vasodilators, principally nitric oxide.^2^ In agreement, in vitro studies in human isolated vessels show that apelin is a potent vasodilator.^3,4^ Apelin binds to a single G-protein coupled receptor (also known as APJ).^5^ The vasodilatation and inotropic actions of apelin at this receptor make it an attractive target for development of new therapies for heart failure^6–9^ and pulmonary arterial hypertension,^10–13^ conditions in which the apelin system is reportedly downregulated, using agonists to replace the missing apelin. A fundamental limitation of many agonists acting at G-protein coupled receptors, such as apelin, is that after stimulating G-protein pathways to elicit a physiological response, the target receptor is internalized^14–17^ and silenced via the β-arrestin pathway.^14^ Furthermore, although apelin activation of the Gαi-protein pathway elicits a protective response in the heart, in the absence of apelin, stretch signals through the apelin receptor are mediated via β-arrestins resulting in detrimental cardiac hypertrophy.^18^ Evidence is emerging that agonists acting at the *same* receptor can be engineered to stabilize different conformational states and selectively stimulate either the G-protein (eg, μ-opioid receptor)^19^ or β-arrestin (eg, angiotensin AT1 receptor)^20^ pathway and function as biased agonists.^20,21^

We hypothesized that an agonist at the apelin receptor designed to display bias toward the G-protein pathway would produce vasodilatation and increased cardiac output with reduced loss of efficacy owing to receptor downregulation. We have simulated apelin and cyclic apelin peptide analogs in aqueous solution and also used homology modeling and molecular dynamics simulations of apelin with its receptor to design ≈105 cyclic peptide analogues. These were tested for inhibition of radiolabelled apelin binding followed by a screening cascade comparing G-protein–dependent versus G-protein–independent pathways to identify a biased agonist, MM07. As proof of principle in first-in-human studies, we show MM07, as predicted, is a more effective vasodilator in human vasculature than apelin and increases cardiac output in rodent by echocardiography.

## Methods

Additional information is available in the online-only Data Supplement. Animal experiments were performed according to local ethics committee (Universities of Cambridge and London) and Home Office (UK) guidelines under the 1986 Scientific Procedures Act.

### Computational Methods

From analysis of molecular dynamics simulations, MM07 (cyclo[1–6]CRPRLCHKGPMPF) was expected to mimic the solution conformation of apelin-13 (NH_2_-QRPRLSHKGPMPF-COOH) and promote a β-turn conformation at the RPRL motif, suggested to be important for initial recognition and binding at the apelin receptor.^22–24^

### Simulation Protocol

The model of MM07 was constructed using the Biopolymer module of the Sybyl 7.3 program^25^ and energy minimized in vacuo with the Amber11 FF99SB force field.^26^ Gromacs version 3.3.1^27-29^ was used for all simulations. Replica exchange molecular dynamics^30^ was used to explore the conformational space of the peptides.

### Homology Modeling of the Apelin Receptor

The modeling template was based on the 2.5 Å resolution crystal structure of the human CXCR4 chemokine receptor. MODELLER9v8 was used to generate homology models of apelin and subsequently refined using molecular dynamics.

### Human Tissue Collection

Human tissues were obtained with informed consent (Papworth Hospital Research Tissue Bank REC08/H0304/56) and experiments performed with local ethical approval (REC05/Q0104/142). Saphenous vein was from 19 patients receiving coronary artery bypass grafts. Heart tissues were from 3 patients undergoing cardiac transplantation.

### Competition Binding Assays

Initial assays were performed in cells expressing the human apelin receptor (CHO-K1-APJ cells; Cerep, Celle L’Evescault, France). In subsequent experiments, homogenate of human left ventricle was incubated with 0.1 nmol/L [Glp^65^,Nle^75^,Tyr^77^][^125^I]apelin-13 and with [Pyr^1^]apelin-13 or MM07 (0.01 nmol/L–100 μmol/L). Nonspecific binding was defined using 1 μmol/L [Pyr^1^]apelin-13. Data were analyzed (KELL package; Biosoft, UK) to obtain the dissociation constant *K*_D_ (concentration of ligand occupying 50% of receptors).

### Cell-Based Functional Assays

β-Arrestin and receptor internalization assays (AGTRL1; DiscoveRx, Fremont, CA) used cells expressing the human apelin receptor. Agonist concentration-response curves were analyzed to determine values of pD_2_ (−log_10_ of the EC_50_ [the concentration of agonist producing 50% of maximum response]) and maximum response (*E*_MAX_) for [Pyr^1^]apelin-13 and MM07.

### Human Saphenous Vein Contraction Study

The predominant effect of apelins in vivo is vasodilatation^2^ and apelin peptides are equi-effective dilators of human endothelium-intact arteries in vitro.^3^ However, removal of the endothelium in saphenous vein unmasks apelin contraction,^3^ which we have exploited as a routine bioassay. Cumulative concentration–response curves to [Pyr^1^]apelin-13 and MM07 (10^−12^ to 10^−7^mol/L) were expressed as a percent of the maximum response to 100 mmol/L KCl. Data were analyzed as described for the cell based assays.

### Analysis of MM07 Signaling Pathway Bias

Data from in vitro assays were analyzed as previously described^31^ to determine the pathway signaling profile for MM07 compared with [Pyr^1^]apelin-13 to show whether MM07 exhibited additional bias for the individual signaling pathways investigated.

### In Vivo Plasma Half-Life of Apelin Peptides

Rats (230–260 g), anaesthetized with 2% isoflurane, were given a single intravenous bolus (600 nmol) of apelin or MM07 (both n=3). Arterial blood samples were taken over the subsequent 30 minutes and plasma concentrations measured using an enzyme immunoassay that detects both peptides, and plasma half-life was determined.

### Systemic Infusions in Rat

Surgical preparation and experimental techniques were as described previously.^32^ After baseline measurements, male Wistar rats (300±25 g body weight) received vehicle (300 μL bolus), then incremental doses of [Pyr^1^]apelin-13 or MM07 (1–300 nmol/300 μL), followed by *S*-Nitroso-*N*-acetyl-d,l-penicillamine (SNAP). Blood pressure (BP) was monitored continuously via an intra-arterial line and echocardiography was used after each bolus to determine changes in heart rateHR, stroke volume, cardiac output (from peak velocity and velocity time interval) and respiratory rate. Rectal temperature was monitored throughout. BP was analyzed using absolute change in mean BP from the baseline taken just before the bolus and area under the curve analysis.

### Human Volunteer Studies

All volunteers (n=12 forearm plethysmography, n=10 hand vein studies) gave written informed consent, and studies adhered to the principles of the Declaration of Helsinki and were approved by the National Research Ethics Service Committee East of England–Cambridge Central (REC 11/EE/0305).

### Forearm Venous Occlusion Plethysmography

[Pyr^1^]apelin-13 and MM07 were infused in 3 incremental doses, 6 minutes each, using the previously determined optimal dose range. The order of peptides was randomized, separated by 20 minutes saline infusion, and the study completed by infusion of sodium nitroprusside (3 µg/min for 6 minutes) or saline. Forearm blood flow (FBF) was measured in both arms and expressed as absolute change in FBF in response to agonists. In a second study (n=4), the effect of repeated (3 doses, each for 8 minutes) infusions of MM07 was determined after a 30 minute saline washout.

### Aellig Hand Vein Technique Study

After baseline measurements, norepinephrine (1–60 ng/min) was infused in 7 minute increments (7.5 mL/h) to achieve a stable constriction of 50% to 80%. [Pyr^1^]apelin-13 or MM07 (4 incremental doses, each for 8 minutes at 7.5 mL/h) were infused 30 minutes later, with measurements taken in the last 2 minutes of each infusion period followed by a 30 minute saline infusion.

### Statistical Analysis

For in vivo animal and human volunteer studies, variables are reported as mean±standard error of the mean. Data were analyzed using 1- or 2-way ANOVA or 2-way repeated measures ANOVA. Post hoc multiple comparisons were made using Sidak’s or Tukey’s post hoc test as appropriate (Graph-Pad Prism; Graph-Pad Software Inc, San Diego, CA). Student’s 2-tailed *t* test was also used as appropriate. Statistical significance was taken at the 5% level.

## Results

### Design and Conformation of MM07

**Figure 1.**
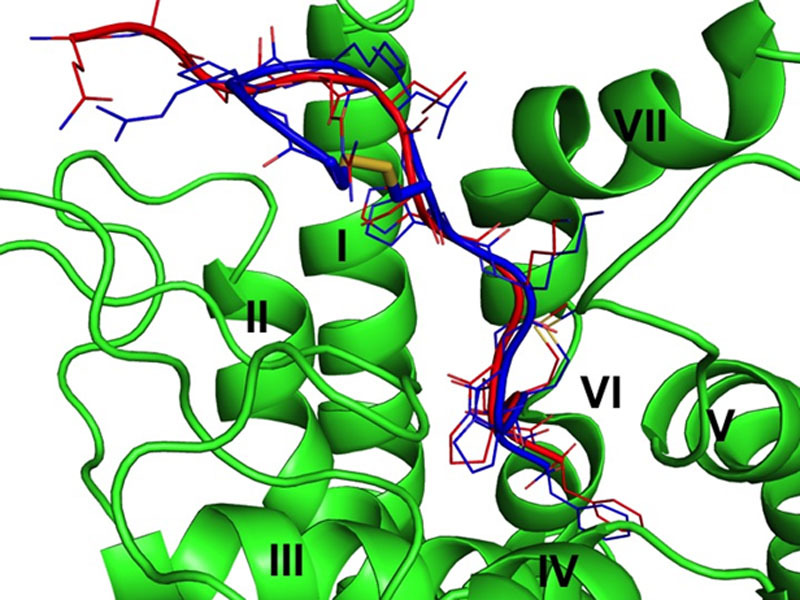
Representation of the overlap between the calculated poses of apelin-13 (red) and MM07 (blue) binding to the apelin receptor. The disulphide bond of MM07 is indicated in stick, the side chains in lines, and the backbone in cartoon representation. The transmembrane helices of the apelin receptor are indicated by roman numerals.

To investigate the receptor bound conformation of MM07, compounds were initially docked into a receptor homology model and the complex refined using molecular dynamics. The apelin and MM07-apelin receptor binding poses are shown in Figure 1 taken from the most representative structure of the principal cluster of the MD simulation trajectory. The results implied sufficient volume was available for the cyclized peptide to occupy a similar binding pose to apelin-13.

### MM07 Binds to the Human Apelin Receptor

MM07 competed with nanomolar affinities for binding of [Glp^65^,Nle^75^,Tyr^77^] [^125^I]apelin-13 to human apelin receptors in CHO-K1 cells (*K*_D_, 300 nmol/L) and human heart (*K*_D_, 172 nmol/L, n=3).

### MM07 Is a G-Protein Pathway Biased Agonist In Vitro

**Figure 2.**
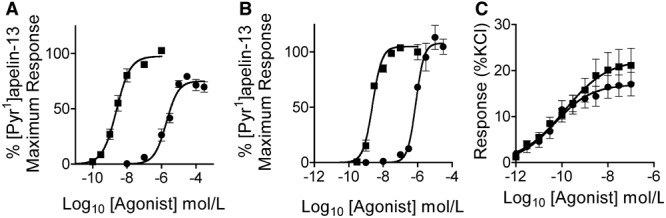
Comparison of in vitro functional activity of [Pyr^1^]apelin-13 () and MM07 (●) in G-protein-independent (**A**) β-arrestin recruitment and (**B**) receptor internalization assays and G-protein-dependent (**C**) saphenous vein contraction assay.

In the β-arrestin (Figure 2A) and internalization (Figure 2B) assays, MM07 (β-arrestin pD_2_=5.67±0.1, n=6; internalization pD_2_=6.16±0.07, n=3) was ≈790- and ≈215- fold less potent than [Pyr^1^]apelin-13 (β-arrestin pD_2_=8.57±0.1, n=6; internalization pD_2_=8.49±0.1, n=3), respectively. However, in the saphenous vein contraction assay, the 2 peptides had comparable potency (pD_2_ values: [Pyr^1^]apelin-13, 9.93±0.24, n=11; MM07 9.54±0.42, n=11) and maximum response (*E*_MAX_ as %KCl: [Pyr^1^]apelin-13, 22%±4%; MM07, 17%±3%; Figure 2C). Comparing the relative effectiveness of MM07 to activate the G-protein pathway, saphenous vein contraction compared with the non-G-protein–dependent β-arrestin and internalization pathways yielded bias factors for MM07 compared with [Pyr^1^]apelin-13 of 1374 and 353, respectively (Table).

**Table.**
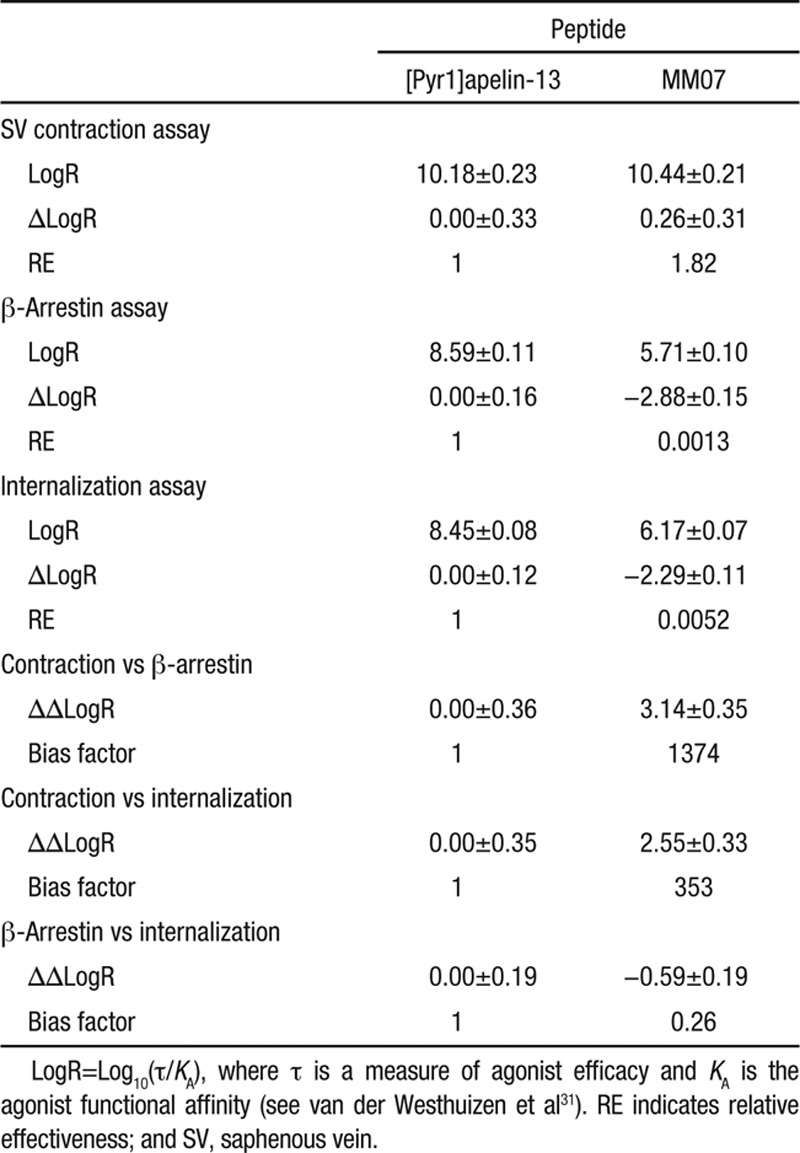
Estimation of G-Protein Pathway Bias for MM07 Compared With [Pyr1]apelin-13 in Saphenous Vein Contraction, β-Arrestin Recruitment, and Receptor Internalization Assays

### Half-Life of [Pyr^1^]apelin-13 and MM07

Plasma half-life of MM07 was 17.4±0.40 minutes compared with 2.3±0.51 minutes for [Pyr^1^]apelin-13 (*P*<0.05, 2-tailed Student’s *t* test).

### MM07 Increases Cardiac Output in Rat

**Figure 3.**
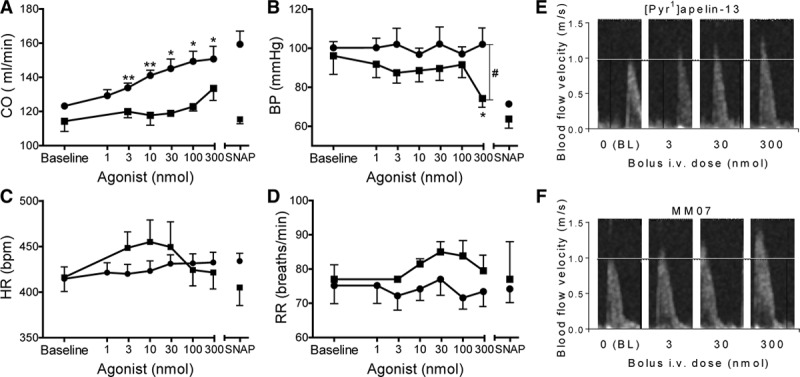
Response to [Pyr^1^]apelin-13 (, n=6) and MM07 (●, n=5) in anaesthetized rats showing changes in cardiac output (CO; **A**), change in blood pressure (BP) from baseline (**B**), heart rate (HR; **C**), and respiratory rate (RR; **D**). Echocardiograms showing an increase in peak velocity across the left ventricular outflow tract after bolus administration of [Pyr^1^]apelin-13 (**E**) and MM07 (**F**). Horizontal line indicates baseline (BL) peak velocity. Significantly different from baseline *P<0.05, **P<0.01 or [Pyr^1^]apelin-13 #*P*<0.05 (ANOVA).

There was a trend to increase in cardiac output with the highest dose of [Pyr^1^]apelin-13, which corresponded to a significant drop in BP (Figure 3A and 3B) and systemic vascular resistance (Figure S3B in the online-only Data Supplement). In contrast, MM07 caused a dose-dependent increase in cardiac output (ANOVA versus baseline, *P*=0.0074; Figure 3A), and although there was a decrease in vascular resistance (Figure S3B), this was without corresponding effects on BP (Figure 3B). Administration of SNAP produced a profound fall in BP in both [Pyr^1^]apelin-13 and MM07-treated groups; however, although cardiac output was significantly increased in response to SNAP in the MM07 group, it was significantly reduced in the [Pyr^1^]apelin-13 group. Neither peptide caused a significant change in heart rate (Figure 3C), respiratory rate (Figure 3D), or temperature (not shown). Both [Pyr^1^]apelin-13 and MM07 increased peak velocity above basal levels (ANOVA versus baseline *P*=0.046 and *P*=0.01, respectively; Figure 3E and 3F).

### MM07 Is a Vasodilator in Human Forearm Venous Occlusion Plethysmography Studies

**Figure 4.**
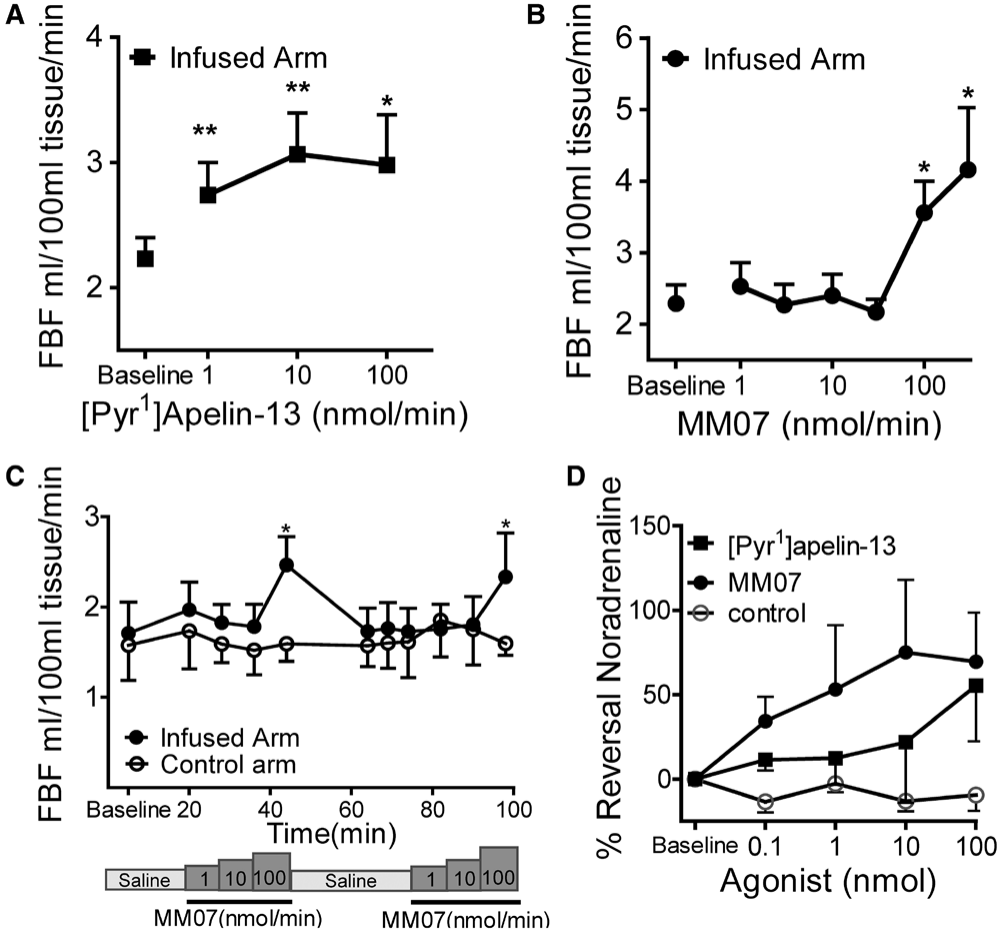
Absolute change in forearm blood flow (FBF) during intrabrachial infusion of [Pyr^1^]apelin-13 (**A**) and MM07 (**B**). **C**, Repeated doses of MM07 produce a significant and reproducible response to 100 nmol/min (*P*<0.05). **D**, Concentration-dependent dilatation in dorsal hand veins after infusions of [Pyr^1^]apelin-13, MM07, and saline control. Significantly different from baseline *P<0.05, **P<0.01 (ANOVA).

[Pyr^1^]apelin-13 (Figure 4A) and MM07 (Figure 4B) produced a significant dose-dependent increase in FBF compared with baseline (ANOVA *P*<0.01). [Pyr^1^]apelin-13 at 100 nmol/min produced an increase in FBF in the control arm. This was not seen with MM07 (data not shown). Importantly, the maximum dilatation to MM07 was approximately double that seen with [Pyr^1^]apelin-13, consistent with a more efficacious action of the biased agonist. Repeated dose–response curves to MM07 produced the same increase in FBF (ANOVA *P*=0.983), which was significant at 100 nmol/min for both; washout was complete and no desensitization was observed (Figure 4C).

### MM07 Reverses Norepinephrine Tone in the Human Hand Vein (Aellig Technique Study)

The dose of norepinephrine required to produce a similar degree of preconstriction (62.3%±3.4%) ranged from 2 to 60 nmol/min. Compared with control data from the same volunteers, there was a significant reversal of norepinephrine constriction to [Pyr^1^]apelin-13 at 100 nmol/min and MM07 at 10 and 100 nmol/min (Figure 4D).

## Discussion

This is the first study to examine the in vivo effects of a novel synthetic biased agonist, MM07, at the apelin receptor in human volunteers.

### MM07 and Peripheral Arterial Tone

[Pyr^1^]apelin-13 (1–10 nmol/min) produced a significant increase in FBF similar to that reported previously for 0.3–3 nmol/min.^33^ At 100 nmol/min, there was no further response but some evidence of dilatation in the control arm, suggesting spillover at this highest dose tested. MM07 caused rapid and significant peripheral arterial dilatation. Although higher concentrations of MM07 were required, there was no evidence at any dose of effects in the control arm, and indeed it is possible that the rapid increase in flow in response to MM07 effectively resulted in dilution of the peptide that may explain the apparent need for higher concentrations in the forearm compared with the hand vein relative to [Pyr^1^]apelin-13. This is supported by the observation that at the 2 higher doses, the % increase in blood flow from baseline was double that of [Pyr^1^]apelin-13, suggesting that MM07 is an effective arteriolar dilator and may be a useful tool compound for use in further studies. Plasma half-life for MM07 in rat was significantly longer than that of [Pyr^1^]apelin-13. Previous studies have reported rapid and prolonged apelin receptor internalization^14,17^ after exposure to endogenous apelin, consistent with rapid removal from the plasma. The longer half-life of MM07 may therefore reflect reduced internalization as indicated by our in vitro assays. Crucially, following MM07, forearm circumference returned to baseline and a second incremental exposure to MM07 produced the same vasodilatory response; no desensitization was seen. This is consistent with our in vitro assays showing MM07 causes reduced internalization compared with [Pyr^1^]apelin-13.

### MM07 and Venous Tone

Responses in resistance and capacitance vessels do not always correlate; we therefore conducted further studies in peripheral veins. After the arterial studies, we repeated the dose ranging for both peptides and performed our definitive studies at a higher dose range than previously reported.^2^ In the human hand vein, MM07 and [Pyr^1^]apelin-13 reversed an established norepinephrine constriction and significantly increased flow in the venous bed with no adverse events observed. In contrast to arterial studies, both peptides were equally effective in the vein comparable to our in vitro contraction assay. The magnitudes of the responses were comparable with acetylcholine and sodium nitroprusside, producing significant reversal of preconstriction.^34,35^ MM07 is therefore an effective peripheral venodilator. The effects of apelin peptides on the central capacitance vessels have not been tested, and further studies are required to determine the in vivo effects of endogenous apelin and related peptides on the systemic vasculature.

### Systemic Effects of MM07 in Rat

Apelin is reported to be the most potent inotropic agent measured in human isolated heart in vitro^3^ with significant effects on cardiac contractility in in vivo animal models,^36,37^ including ischemic cardiomyopathy^38^ and myocardial injury.^39^ The hemodynamic goals in patients with ventricular failure secondary to pulmonary hypertension are decreased pulmonary vascular resistance and augmented cardiac output, which may potentially be achieved with an apelin agonist. Importantly, in heart failure patients, systemic infusion of [Pyr^1^]apelin-13 caused a beneficial peripheral and coronary vasodilatation with increased cardiac output.^33^ These actions were sustained in the presence of angiotensin II and sodium depletion.^40^ To explore the systemic effects of MM07 safely, we used hemodynamic measurements and echocardiography in rats. Echocardiography is the mainstay of the noninvasive assessment of cardiac function in rodent models and has the advantage of clear translation to humans in whom the same parameters are used. In our study, [Pyr^1^]apelin-13 augmented cardiac output that was accompanied by significant hypotension, whereas MM07 caused a significant increase in cardiac output without any evidence of positive chronotropy or hemodynamic instability, and the inotropic effects were comparable with commonly used inotropes, such as dobutamine, milrinone, and isoprenaline.^41–43^ Some of the apparent cardiac effect of [Pyr^1^]apelin-13 may be attributable to a reduction in afterload secondary to peripheral vasodilatation, shown by the drop in BP and systemic vascular resistance (Figure S3B). In contrast, MM07 did reduce vascular resistance (though to a lesser extent than [Pyr^1^]apelin-13), but had no effect on BP or heart rate; we hypothesize that this may be as a result of an additional load-independent inotropic effect. In support of our hypothesis, in isolated perfused rat heart and paced human atrial tissue, apelin caused a sustained increase in contractility,^3,36^ independent of preload and autonomic reflexes. This is predicted to occur by increasing the calcium sensitivity of the contractile machinery via activation of myosin light chain kinase.^44^

### Apelin Agonists in Cardiovascular Disease?

Data suggest that activating the apelin pathway may be valuable in conditions, such as heart failure and pulmonary arterial hypertension. After an initial rise in the early stages of heart failure, plasma levels of peptide tend to decrease in later stages.^6–8^ Although there is downregulation of the apelin receptor pathway in the human heart,^6,9^ the favorable systemic actions of apelin on peripheral vasodilatation and cardiac output remain.^40^ In patients with pulmonary arterial hypertension, >70% have reduced bone morphogenetic protein receptor-2.^45^ Disruption of a bone morphogenetic protein receptor-2–mediated transcriptional complex between PPARγ and β-catenin reduced apelin levels in pulmonary arterial endothelial cells from pulmonary arterial hypertension patients,^10^ and apelin deficiency also causes proliferation in smooth muscle cells.^11^ Apelin-null mice developed more severe pulmonary hypertension compared with wild-type when exposed to chronic hypoxia,^12^ and crucially injections of apelin attenuated myocardial hypertrophy in the monocrotaline-treated rat.^13^ We therefore speculate that apelin agonists, such as MM07, that may have a sustained effect at the apelin receptor would be beneficial in these conditions.

### Perspectives

The results suggest that agonists acting at the apelin receptor can preferentially stimulate the G-protein pathway over β-arrestin, which can translate to improved efficacy in the clinic. The use of biased agonists to selectively stimulate vasodilatation and inotropic action but avoid activating the β-arrestin–dependent stretch response within the heart^18,46^ represents a new therapeutic strategy in the treatment of cardiovascular disease.

## Sources of Funding

We acknowledge the Wellcome Trust Programmes in Translational Medicines and Therapeutics (085686) and in Metabolic and Cardiovascular Disease (096822/Z/11/Z), the British Heart Foundation PG/09/050/27734, the Medical Research Council, the Pulmonary Hypertension Association, and the National Institute for Health Research Cambridge Biomedical Research Centre.

## Disclosures

None.

## Novelty and Significance

### What Is New?

- We report the discovery and first in human in vivo characterization of MM07, a biased agonist for the apelin receptor.

### What Is Relevant?

- The apelin system is downregulated in pulmonary arterial hypertension and heart failure; therefore. targeting the apelin receptor using novel agonists is an attractive therapeutic strategy.

### Summary

Our results suggest that apelin agonists that preferentially stimulate G-protein over β-arrrestin pathways could translate into improved clinical efficacy by selectively stimulating beneficial vasodilatation and inotropic actions but avoiding detrimental β-arrestin-dependent pathways, such as the stretch response in the heart.
